# A facile co-crystallization approach to fabricate two-component carbon dot composites showing time-dependent evolutive room temperature phosphorescence colors†

**DOI:** 10.1039/d1na00362c

**Published:** 2021-07-23

**Authors:** Jian Qu, Xin Zhang, Shuyan Zhang, Zhongjie Wang, Yejian Yu, Huajun Ding, Zhiyuan Tang, Xiangjun Heng, Ruiqi Wang, Su Jing

**Affiliations:** School of Materials Science and Engineering, Yancheng Institute of Technology Yancheng 224051 PR China xinzhang@ycit.edu.cn; School of Chemistry and Chemical Engineering, Southeast University Nanjing 211189 China; School of Chemistry and Molecular Engineering, Nanjing Tech University Nanjing 211816 P. R. China sjing@njtech.edu.cn

## Abstract

Time-dependent evolutive afterglow materials can increase the security level by providing additional encryption modes in anti-counterfeiting and data encryption. The design of carbon-based materials with dynamic afterglow colors is attractive but formidably challenging. In this study, a facile two-component co-crystallization strategy is designed for the first time to obtain N,S-co-doped carbon dots@isophthalic acid (CDs@IPA) and N,S-co-doped carbon dots@melamine (CDs@MA). CDs@IPA and CDs@MA all exhibiting time-dependent evolutive RTP colors from orange *via* yellow to green over 1 s, especially that the green afterglow time of CDs@IPA can reach 6 s (*τ*_avg_ = 582 ms). Studies show that the time-dependent RTP colors originated from two primary emissive centers, low-energy emission of CDs and high-energy emission of host matrix activated by CDs. Due to their distinguishable RTP colors with differentiated lifetimes, the ratios of two RTP emissive bands changed with time during the decay process, resulting in the continuous RTP colors variation in real-time. This two-component carbon dot-based co-crystallization strategy may open a new avenue for the development of time-dependent afterglow color materials.

## Abstract 2

Time-dependent evolutive RTP from N,S-doped carbon dots-based co-crystallizations.

## Graphical abstract

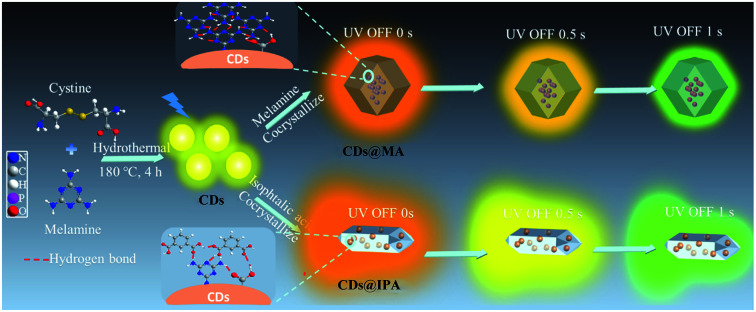

## 1. Introduction

Recently, room temperature phosphorescence (RTP) materials with a long lifetime have received widespread attention due to their potential applications in the fields of information storage,^1^ time-resolved bioimaging,^2^ data encryption^3^ and multimodal anticounterfeiting.^4^ A variety of RTP materials can be classified into two categories, noble metal-containing organometallic^5^ and pure organic compounds,^6^ which have been extensively studied. Most of them, however, just showed a single emission color during their emitting lifetime process and were applied practically by a feat of the intensity changes over time, rather than color evolution, resulting in limited sensitivity and accuracy. If the long-persistent luminescence emissions were time-dependent and their corresponding colors could vary with time, it would endow the materials with richer encryption modes in message protection, definitely, further improving the security level significantly.^7^

Time-dependent color evolution system featured with the dynamic ratio changes of the afterglow emissive bands with time. Multiple distinguishable emissive centers, different long lifetimes and overlapping excitation wavelengths are thought to be the basis for such properties in these materials. However, it is a formidable challenge to integrate the above elements for the construction of versatile time-dependent platforms. Up to now, three scattered examples have been demonstrated, and all of them are pure organic crystals or amorphous polymers, combining with thermally-activated delayed fluorescence (TADF) and RTP, with comparable but distinct decay rates. For example, Chi and co-workers discovered the first example with time-dependent afterglow colors from dibenzofuran derivatives in 2019.^8^ Yang's group reported organic crystals of biphenyl bridged carbazolyl derivatives, which exhibit time-dependent afterglow colors from blue to the orange over 1 s.^7^ Yuan *et al.* further developed a colorful, excitation and time-dependent system based on semi-rigidified, hydrogen-bonded, and oxygen barrier characteristics in amorphous polymers.^9^

Carbon dots take superiority in low cytotoxicity, facile preparation, rich source, high photostability and tunable optical properties.^10^ Therefore, it is highly desirable to develop a new type of carbon dot-based afterglow material. It is widely recognized that the ingenious combination of the different long lifetimes and multicolor afterglow emissive centers is the key to realize the time-dependent afterglow performance. To pursue a long afterglow lifetime of CDs, many studies have highly recommended the following three conditions:^11^ (1) efficient spin–orbit coupling and production of triplet excited states from C=O and C=N relevant moieties. (2) A favorable intersystem crossing (ISC) process for further production of the excited triplet state by doping heteroatoms, such as N, P, S, Se and halogens. (3) A stable lowest triplet state (T_1_) and restrained nonradiative transitions *via* confining the motion of the groups on CDs with various host matrices (*e.g.* polyurethane, layered double hydroxides, urea, silica gel, biuret, KAl(SO_3_)_2_·*x*H_2_O, NaCl and zeolites). Recently, unique CD-based RTP materials with ultralong lifetime, multicolor and excitation-dependent properties have been achieved by means of these strategies.^12^ However, as another critical factor of time-dependent afterglow, the distinguishable afterglow colors from multiple emission centers of composite's response to the same excitation are rarely studied up to now.

Herein, we first developed a facile co-crystallization approach to fabricate two-component CD-based time-dependent RTP color composites, CDs@IPA and CDs@MA. Among them, the CDs, serving as not only crystallization additives but an orange RTP emission center, were integrated into hydrogen bonding-rich isophthalic acid (IPA) or melamine (MA) host, which exhibited green or bluish-green RTP emission. These CDs@IPA and CDs@MA made by following a systematic strategy exhibited time-dependent evolutive RTP colors from orange *via* yellow to green over 1 s, especially that the green afterglow time of CDs@IPA can reach 6 s (*τ*_avg_ = 582 ms), after ceasing a 365 nm UV source. The results confirmed that the strengthened hydrogen bonds between CDs and host matrix-forming during the co-crystallization process, along with the encapsulated structure, endowed CDs with RTP properties, and also that observed afterglow was actually a mixture of two different RTP emissions from CDs gust and CDs activated IPA/MA host optical centers. Because of the different decay rates of the RTP emission peaks, the relative strength of these two long-lived emission bands changed with time, resulting in the formation of the time-dependent evolutive RTP colors.

## 2. Experimental

### 2.1 Materials

Melamine (MA), l-cystine, isophthalic acid (IPA), CaCl_2_, isophthalaldehyde and cyanuric acid were purchased from Aladdin Ltd. (≥99%, Shanghai, China). Dialysis bags (molecular weight cut off 3000) were acquired from Green Bird Science & Technology Development Co. (Shanghai, China). Deionized water was widely used throughout the experiments.

### 2.2 Equipment and characterization

X-ray photoelectron spectroscopy (XPS) analysis on CDs was carried out with a Thermo ESCALAB 250 X-ray photoelectron spectrometer (USA). The binding energy scale was calibrated by the standard value of C 1s at 284.6 eV. The morphology and particle size of the synthesized CDs, CDs@MA and CDs@IPA were collected using the JEM-2100F transmission electron microscope (TEM, JEOL Ltd., Japan). The FT-IR characteristic absorption bands in the sample surface were recorded on a Bruker Vector 22 FT-IR spectrophotometer using KBr pellets. Powder X-ray diffraction (XRD) data were measured using a Bruker D8 discover X-ray diffractometer (Bruker, Germany). The Raman spectrum was collected using a Thermo Scientific DXRxi Raman spectrometer (USA) with an excitation wavelength at 532 nm. The heights of CDs were characterized by atomic force microscopy (AFM) using a Brucker Dimension Edge in the ScanAsyst mode under ambient conditions. The zeta potential of the CDs was determined using Malvern Zetasizer (Nano ZS, England). UV-vis absorption spectra were recorded on the UV-2450 spectrophotometer (Tokyo, Japan). The fluorescence (FL), phosphorescence (Phos) emission and excitation spectra were measured on a HITACHI F-7000 under ambient conditions. FL and Phos lifetimes were determined using a FluoroLog 3-TSCPC (Horiba Jobin Yvon Inc., Japan). The absolute quantum yield was obtained using a quantum yield accessory, including an integrating sphere (Horiba Jobin Yvon Inc. Japan). The time-resolved luminescence spectra were acquired on an Edinburgh FLS980 instrument. The confocal fluorescence images in a green channel (520–560 nm) were collected by exciting at 405 nm, on a Zeiss LSM880NLO (2 + 1 with BIG) confocal microscope system. Photographs and videos of RTP emission recorded with an iPhone 8 under different excitation by hand-held UV lamps (275 nm@18 mW, 310 nm@15 mW, 365 nm@3 W and 395 nm@3 W, Taoyuan, China).

### 2.3 Synthesis of CDs

These CDs were synthesized by a traditional hydrothermal approach with melamine as the nitrogen source and l-cystine as the sulfur source. Typically, the mixtures containing 0.19 g melamine and 0.21 g l-cystine were dissolved in 30 mL of deionized water. After 15 min of ultrasonic treatment, the formed solution was transferred into a 50 mL Teflon-lined autoclave and heated at 180 °C for 4 h, then automatically cooling down to room temperature. Subsequently, the acquired orange solution was collected through centrifugation at 10 000 rpm for 20 min. After storing under 4 °C for 24 h, the resulting CDs were further centrifuged to remove the excess raw materials. Finally, the as-prepared CDs solution was dialyzed in a dialysis bag (1000 Da) for 48 h and solidified *via* freeze-drying.

### 2.4 Synthesis of CDs@IPA and CDs@MA

0.15 g IPA or 0.11 g MA solution was first diluted with 5 mL DI water and mixed with 10 mL CDs solution (1.5 mg mL^−1^). After being heated in an oil bath at 130 °C for 30 min, the turbid solution became clear. Then, it was cooled to room temperature naturally when light yellow needle crystals (square crystal for MA) were obtained. After washing twice with DI water and dried in the air, the final needle-like CDs@IPA and blocky CDs@MA were collected.

### 2.5 Synthesis of CDs@CaCl_2_, CDs@isophthalaldehyde and CDs@cyanuric acid

They followed similar synthesized processes as the preparation of CDs@IPA, except that the 0.15 g IPA was replaced by the 0.21 g CaCl_2_, 0.18 g isophthalaldehyde and 0.18 g cyanuric acid, respectively.

## 3. Results and discussion

### 3.1 Design concept of CDs@IPA and CDs@MA

**Fig. 1:**
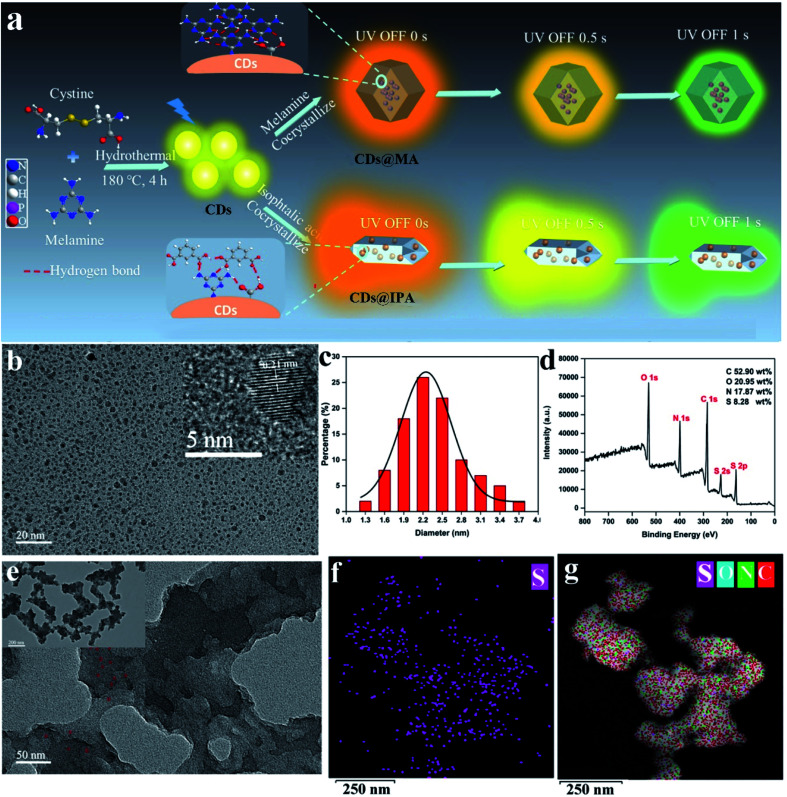
(a) Scheme for the synthesis and luminescence properties of CDs@MA and CDs@IPA composites. (b) TEM image of CDs. Inset: HR-TEM lattice fringe image of CDs. (c) Size distribution of CDs. (d) XPS survey spectrum of CDs. (e) TEM images of CDs@MA. The EDX maps of (f) S and (g) C, N, S, O elements in CDs@MA.

To realize the distinguishable multiple emission centers with comparable but different decay rates, we adopted a host–guest strategy, in which low-energy RTP emissive CDs were employed as a guest phase, and a high-energy RTP emissive matrix with different RTP colors and lifetimes was utilized as a host phase. Meanwhile, the effective excitation of the host matrix should be guaranteed under the excitation wavelength of the CDs. The N,S-co-doped strategy was chosen based on the fact that it could easily replace carbon atoms around the defect sites and provide state density, emission trap state as well as spin–orbit coupling enhancement, contributing to the redshift of emission, the improvement of the photoluminescence quantum yield and the facilitation of the ISC process.^13^ MA, which possesses a highly symmetric structure, took a role as a superb candidate for H-bonding and a critical bridge between CDs and host matrices in these co-crystallized systems.^14^ It's surrounding on the surface of CDs, might provide suitable binding sites for self-assembly with other MA molecules or aromatic acids molecules from the matrices through strong H-bonding and π–π stack, to form the compact encapsulation of CDs.^15^ It is favorable for the highly efficient production of RTP from CDs. Notably, MA also could behave as highly efficient bluish-green or green ultralong phosphorescence when crystallized with itself or IPA.^16^ Thus, the distinguishable colors and lifetimes of RTP from CDs and matrix emissive centers may be incentive to realize dynamic RTP (Fig. 1a).

### 3.2 Characterization of CDs, CDs@IPA and CDs@MA

Since the phosphorescent behaviors were highly related to their microstructures, the morphology, chemical compositions and the functional groups at the surface of CDs were thus analyzed using transmission electron microscopy (TEM), atomic force microscopy (AFM), X-ray photoelectron spectroscopy (XPS) and Fourier transform infrared (FT-IR), respectively. As shown in Fig. 1b, the uniform and quasi-spherical-shaped nanoparticles with an average size of 2.5 nm were well separated from each other (Fig. 1c). Moreover, the HR-TEM image revealed a typical lattice spacing of 0.21 nm (Fig. 1b, inset), which fitted well with the (100) facet of graphite.^17^ The topographic heights were mainly in the range of 1.5–2.5 nm, suggesting that most of the CDs comprise 4–7 layers of graphene sheets (Fig. S1†).^18^ The XPS analysis showed that CDs mainly consisted of C, N, O and S with weight percentages of 52.90 wt%, 17.87 wt%, 20.95 wt% and 8.28 wt%, respectively (Fig. 1d). The high-resolution spectrum of C 1s exhibited four typical peaks at 288.5, 287.6, 285.7 and 284.5 eV, corresponding to C=O/C=C, C–N, C–O and C=C/C–C groups, respectively (Fig. S2a†).^19^ The N 1s peaks at 401.9, 400.8 and 399.5 eV illustrated most of the nitrogen element present in the form of graphite, pyrrole and amine (Fig. S2b†).^20^ The O 1s signals at 533.8, 532.1 and 531.1 eV confirmed the presence of O–C=O, C–O, C=O and C–O (Fig. S2c†).^21^

The binding energy peaks of S 2p at 164.2 and 163.1 eV were ascribed to S=O and C=S bonds,^21^ and another two peaks around 167.6 and 168.2 eV could be assigned to C–SO_2_ and C–SO_3_, respectively (Fig. S2d†).^13*b*^ In the FT-IR spectrum, the broad absorption peaks between 3100 and 3500 cm^−1^ were assigned to NH_2_ and OH vibrations, retained from melamine and l-cystine precursors (Fig. S3†).^22^ The characteristic peaks located at 1655, 1623 and 1550 cm^−1^ originated from the stretching vibration of C=O, C=N and C=C, respectively.^23^ Three weaker peaks appeared at 1398, 1138 and 1024 cm^−1^, attributed to stretching vibrations of C–N, C–O and C–S.^24^ Furthermore, the characteristic peak appearing at 1437 cm^−1^, associated with the aromatic triazine ring stretching vibration of MA molecules, suggested that MA molecules were reserved on the surface of CDs.^25^

Moreover, the zeta potential of the above CDs was detected as +13.55 mV, which is closely associated with plenty of NH_2_ functional groups surrounded on their surfaces.^10*e*^

All these results clearly verified that the quasi-spherical CDs were composed of crystallized sp^2^ carbon core and surrounded by abundant functional groups on their surfaces, such as MA, COOH, OH, NH_2_, S=O, C=N and so on. It endowed these nanomaterials with high solubility, a considerable amount of potential luminescence centers as well as adequate binding sites for IPA and MA through strong intermolecular hydrogen bonds.

**Fig. 2:**
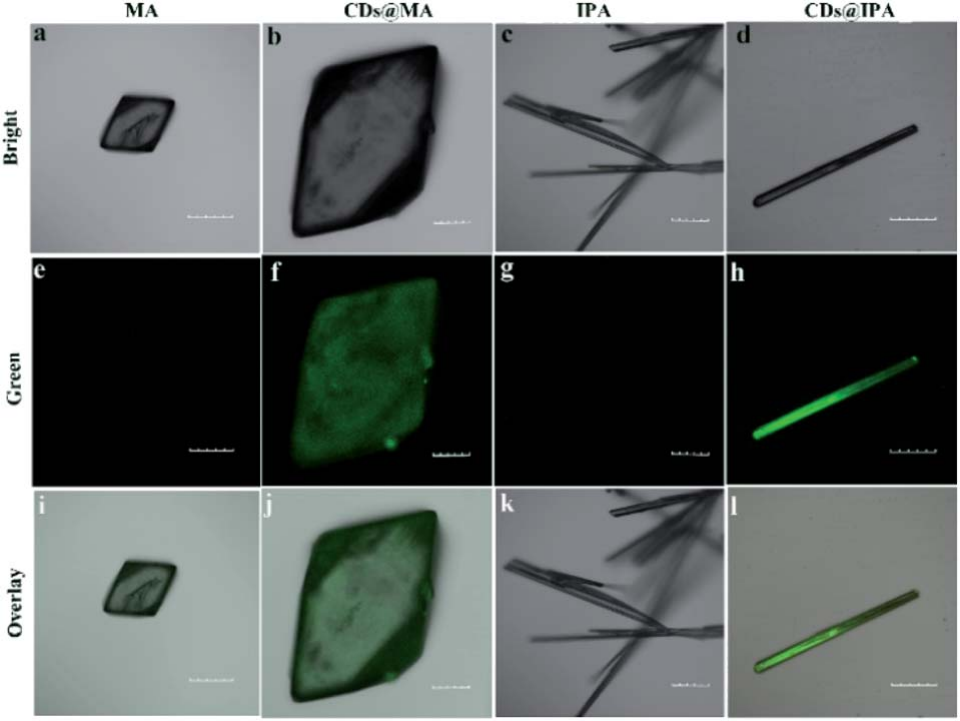
Fluorescence confocal microscopic images of (a, e and i) MA, (b, f and j) CDs@MA, (c, g and k) IPA and (d, h and l) CDs@IPA crystals. The green channel was collected in the range of 520–560 nm (*λ*_ex_ = 405 nm). Scale bar: 100 μm.

The interior of the as-prepared CDs@host composites was also analyzed. The TEM images showed that the resulting CDs@IPA composite displayed a sheet-like morphology (Fig. S4†) and the CDs@MA composite displayed a ginger-like morphology (Fig. 1e). From these TEM micrographs, it also could be seen that a large number of dark dots were highly dispersed on supports. Combining the results of EDS mapping (Fig. 1f and g), it was demonstrated both CDs were well dispersed within the corresponding host matrix. The XRD pattern (Fig. S5†) and Raman spectra (Fig. S6†) of the bare matrix crystals and CDs@MA/CDs@IPA crystals were almost identical, demonstrating that matrix crystals composed of MA or IPA were well preserved upon embedding in CDs. Besides, further confirmation of the location of the CDs within their organic host matrices in crystals was recorded using confocal fluorescence microscopy (CFM) (Fig. 2). Considering the host phases, MA or IPA, were not luminescent in a green channel under 405 nm excitation (Fig. 2e and g), any luminescence observed should be derived from the CDs (Fig. 2f and h). The data also clearly demonstrated that the CDs were well embedded in the matrix, although a little non-uniform green fluorescence intensity was presented in all zones, resulting from the face-specific additive binding and culture environments of the single crystal.^11*a*^

These results evidenced the CDs were well embedded in the IPA and MA matrices after the procedure of co-crystallization. Such an encapsulated structure may benefit the formation of stable T_1_ specials and even ultralong RTP properties by confining the groups on CDs, barring the atmospheric O_2_ and H_2_O to suppress the nonradiative decay process.

### 3.3 Photophysical properties of CDs, CDs@IPA and CDs@MA

**Fig. 3:**
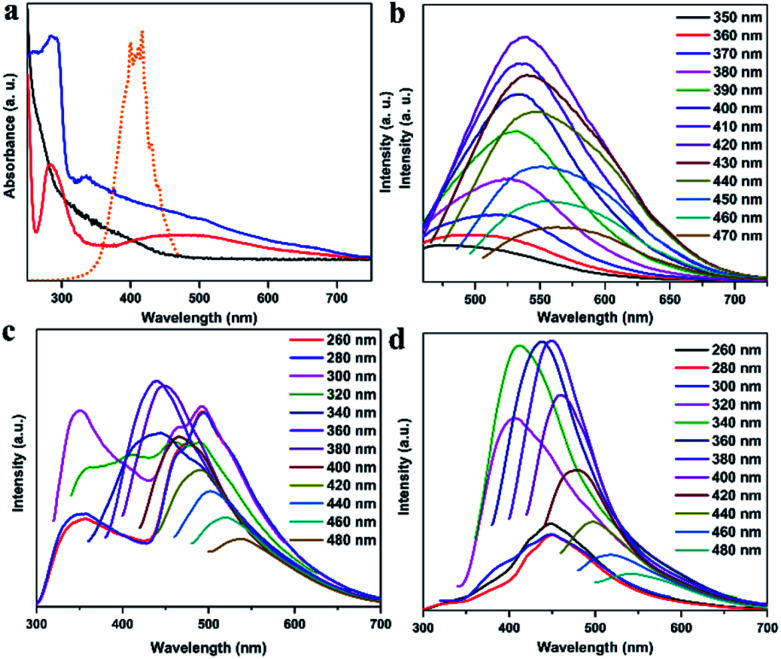
(a) UV-vis absorbance spectra (black line for CDs, blue line for CDs@IPA and red line for CDs@MA) and FL excitation spectrum of CDs (orange line). FL emission spectra of (b) CDs, (c) CDs@IPA and (d) CDs@MA under different excitation wavelengths.

**Fig. 4:**
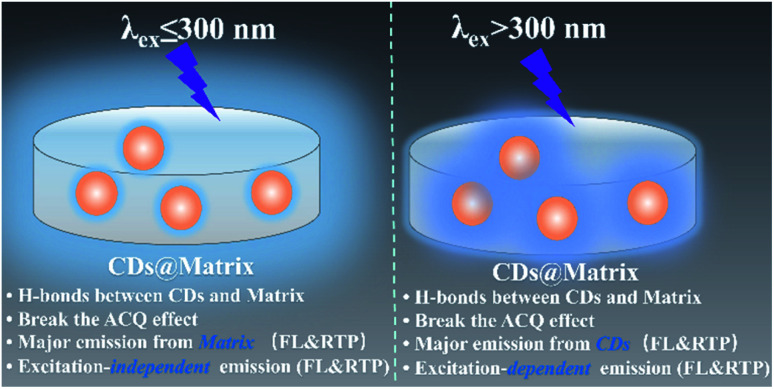
Schematic diagram of the generation of solid FL and RTP emission.

The photophysical properties of CDs, CDs@IPA and CDs@MA were systematically investigated. The UV-vis spectra of CDs aqueous dispersion in Fig. 3a indicated a typical absorption peak at 288 nm and a broad absorption band spanning from about 290 to 500 nm, stemming from the π–π* transition of aromatic sp^2^ domains and n–π* transitions of C=O/C=N bonds, respectively.^26^ Notably, the optical density and the absorption range of both CDs@host composites were greatly enhanced and expanded, owing to the changes on the surface of CDs after co-crystallization with IPA and MA, and making it more efficient energy absorption in a broader range (Fig. 3a). Afterward, the as-obtained CDs dispersion exhibited a typical excitation-dependent emission property in FL emission spectra, and its maximum emission intensity was centered at 542 nm (absolute FL QY of 13.7%, *τ*_avg_ = 5.85 ns), accompanied with bright yellow fluorescence under 420 nm optical excitation (Fig. 3b and S7a†). However, CDs didn't show any solid fluorescence, implying that the aggregation-induced quenching effect might have occurred.^27^ Moreover, the FL excitation spectrum of CDs, just located at the absorption region of C=O/C=N moieties, was responsible for the FL emission (Fig. 3a). Interestingly, in FL emission spectra of CDs@IPA and CDs@MA, co-crystallized samples exhibited an excitation-dependent emission property when the exciting wavelength was above 300 nm, and an excitation-independent emission property when the exciting wavelength was below 300 nm. Moreover, these samples had a longer emission wavelength when excited below 300 nm than at 300–320 nm. On the basis of the above results, it is reasonable to deduce that such a co-crystallized system might be composed of two primary luminescent components, excitation-dependent CDs and excitation-independent IPA/MA matrix activated by CDs (note that the residual MA molecules orientated from the CD surface favored the formation of H-bond with IPA/MA matrix). The emission dominant wavelength was highly based on the effective excitation of CDs or matrix. In these solid composites, the emission from the CDs was obtained, owing to the broken aggregation-induced self-quenching effect, which was considered to be the interaction between confined CDs and the carboxyl/amino groups of IPA/MA, making CDs dispersedly embedded in the corresponding matrix. The excitation-independent emissions centered at 493 (CDs@IPA) and 449 nm (CDs@MA) differed from those of the corresponding pristine IPA and MA (Fig. 3c, d and S8†), under the excitation wavelength less than 300 nm, confirming that such FL signals came from CD-activated IPA/MA components (Fig. 4). Compared with CDs, both CDs@IPA and CDs@MA had a prominent blue FL emissive shift (∼50 nm), and a slight decrease in average FL lifetimes (4.14 ns for IPA composite, 4.42 ns for the MA composite) (Fig. S7†).

**Fig. 5:**
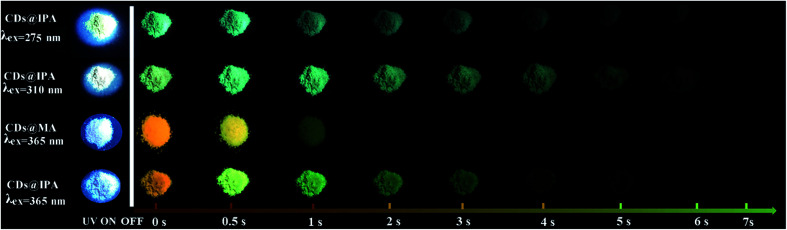
Photographs of CDs@IPA and CDs@MA taken before and after switching off the corresponding excitation in the scale from 0 to 7 s.

The blue shift of emissive peaks was due to the strong H-bonding effect between CDs and host matrix, which altered the electronic structure, luminescent centers and defect state of CDs.^28^ Remarkably, afterglow colors dynamically varied with time from the as-prepared CDs@IPA and CDs@MA materials could be easily recognized by the naked eye under ambient conditions, after removing the corresponding excitation source. In detail, at first, CDs@IPA glowed with a bright orange afterglow, after turning off a 365 nm UV light. Rapidly, it evolved its afterglow colors from initiation orange *via* yellow, to green within 1 s. Subsequently, the ultimate green persistent luminescence faded away until another 6 s (Fig. 5). However, when the 365 nm UV-light was replaced by a 310 nm analogue, the identical dynamic afterglow colors with shorter duration were left to be observed, but the colors just turned from yellow to green with the passage of time after ceasing the irradiation (Fig. 5). Furthermore, when the excitation source was replaced by a 275 nm deep UV-light, the invariable blush green afterglow was captured (Fig. 5). It might be ascribed to the shorter afterglow lifetime or inefficient excitation of multicomponents. Although the afterglow colors faded relatively quickly in the CDs@MA system, there was also a similar evolutive afterglow phenomenon that was captured by the naked eye after switching off a 365 nm UV light (Fig. 5). The average lifetimes under the above different excitation wavelengths, listed in Table 1, were calculated based on the following eqn (1):

1 *τ*_avg_ = Σ*α*_*i*_*τ*_*i*_^2^/Σ*α*_*i*_*τ*_*i*_

**Table 1:** The RTP lifetimes of CDs@IPA and CDs@MA under different excitation wavelengths

*λ*_ex (nm)_	CDs@IPA		CDs@MA	
	*λ*_em (nm)_	*τ*_avg (ms)_	*λ*_em (nm)_	*τ*_avg (ms)_
275	509	688	454	198
310	511	621	458	147
365	550	578	550	89

To the best of our knowledge, this is the first example of a time-dependent afterglow system developed using co-crystallization engineering based on carbon dots.

**Fig. 6:**
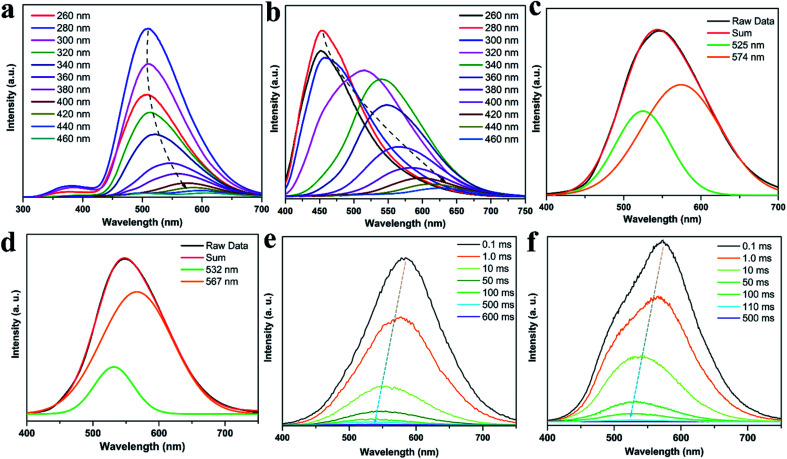
(a) RTP emission spectra of CDs@IPA and (b) CDs@MA under different excitation wavelengths. (c) The RTP emission spectra of CDs@IPA and (d) CDs@MA at *λ*_ex_ = 360 nm, and the Gaussian fitted by two emission peaks. (e) Time-resolved emission spectra of CDs@IPA and (f) CDs@MA.

To gain insights into such unique RTP properties of CDs@IPA and CDs@MA, their afterglow decays were determined. The RTP spectra showed that their emission maxima were located at 509 (CDs@IPA) and 454 nm (CDs@MA) when excitation wavelength was below 300 nm, then red-shifted from about 512 to 605 nm (CDs@IPA) and 516 to 650 nm (CDs@MA) in response to the excitation wavelength changes from 320 to 460 nm (Fig. 6a and b). Such a large shift (100–200 nm) was rarely reported previously, which implied that the existence of multiple S_1_ states and T_1_ states in this system. The RTP lifetimes of CDs@host composites were measured by their time-resolved spectra were measured by their time-resolved spectra excited at 360 nm. All decay spectra collected at about 546 nm, could be fitted by a tri-exponential function with three components (Fig. S10 and Table S1†). These results confirmed that there were multidecay channels responsible for the RTP emission. The average lifetimes were calculated to be 582 (CDs@IPA) and 91 ms (CDs@MA), respectively.

**Fig. 7:**
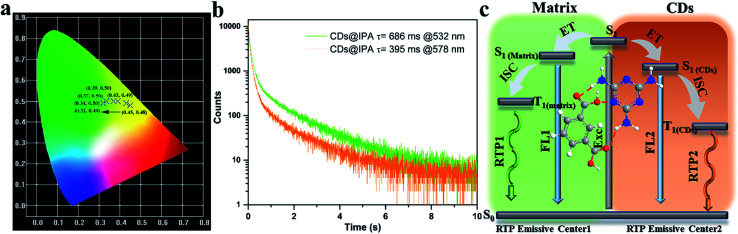
(a) CIE coordinates of time-dependent colors of CDs@IPA. (b) Corresponding RTP decay profiles of CDs@IPA. (c) The proposed mechanism of time-dependent evolutive RTP colors for the CDs@host matrix.

To make further clarifications on time-dependent RTP properties, more in-depth investigations were carried out. First, RTP spectra of CDs@IPA and CDs@MA collected at 360 nm excitation wavelength were analyzed by fitting with Gaussian function.^29^ As shown in Fig. 6c and d, all these spectra could be fitted with two Gaussian peaks with significantly different regions of emission peaks and phosphorescence intensity. Taking the CDs@IPA system, for example, its corresponding fitted two simple Gaussian peaks located at 574 and 525 nm (value difference *ca.* 49 nm) were consistent with their starting and final RTP colors. These dates were further verified by the delayed emission studies. As shown in Fig. 6e and f, under a delay time of 0.1 ms, CDs@IPA and CDs@MA exhibited a highly similar PL profile to that of their fitted Gaussian peaks, with maxima at around 578 and 574 nm, respectively. Their associated orange RTP emission lifetimes were calculated to be 395 (578 nm, CDs@IPA) and 81 ms (574 nm, CDs@MA), implying both of them originated from the same emitted state from CDs, but with different types and strength of hydrogen bonds in the matrix environment. Subsequently, with the delay time controlling from 1 to 600 ms, the delayed emissions of CDs@IPA and CDs@MA significantly blue-shifted within 100 ms, and then gradually reached stable positions at 532 and 527 nm, respectively, accompanying with the clearly reduced intensity. Their associated green RTP emission lifetimes were extended to 686 (532 nm, CDs@IPA) and 101 ms (527 nm, CDs@MA) (Fig. 7b, S11 and Table S1†). The long lifetimes at 532 and 527 nm, were mainly ascribed to the activated IPA/MA host matrices, which increased intermolecular interaction and separation between IPA or MA molecules by the embedded CDs. Notably, all fitted or experimented two simple peaks kept the same rule that the low energy bands have the stronger emission intensity but with shorter RTP lifetimes. On the contrary, the higher energy band with relatively weaker emission intensity obviously showed comparatively longer RTP lifetimes. Therefore, it could be reasonably speculated that observed afterglows of the CDs@host matrix were actually a mixture of two different RTP emissive peaks (578 and 532 nm for CDs@IPA, 574 and 527 nm for CDs@MA) from guest and host optical centers, but the peaks at 578 or 574 nm represented a major contribution at the beginning based on the relative emissive intensity. However, due to the relatively quicker decay rate of the former emissive peaks, the relative strength of these two long-lived emissive bands changed with time, until the RTP peaked at 532 nm (CDs@IPA) or 527 nm (CDs@MA) gradually occupied the main status after the UV-light was switched off for 1 s, resulting in the formation of the time-dependent RTP colors. Correspondingly, for CDs@IPA, the Commission International del'Eclairage (CIE) coordinates evolved from (0.45, 0.48) to (0.37, 0.50) and then to (0.32, 0.49) (evolved from (0.41, 0.48) *via* (0.39, 0.49) to (0.30, 0.46) in the CDs@MA system), agreeing well with the naked-eye observation (Fig. 7a and S12†). Taken all these results together, we reasoned that there might be two primary emissive centers among these CD-based host–guest systems for colorful RTP (Fig. 7c). These distinguishable RTP intensities cooperated well with the inverse tendency of RTP lifetime together, triggering such impressive orange to yellow and then to green evolutive RTP colors. Moreover, to justify such a hypothesis, a set of contrasting experiments were further conducted using three kinds of host matrices, CaCl_2_, isophthalaldehyde and cyanuric acid to co-crystallize, instead of MA or IPA (Fig. S13†). As expected, no obvious time-dependent RTP colors were observed, indicating the compulsory condition of the powerful H-bonds impact as well as suited self-afterglow performance for the specific matrix.

Since commonly acknowledged that the energy gap (*Δ*_EST_) between the lowest S_1_ and lowest T_1_ could be estimated through their low-temperature fluorescence and phosphorescence spectra,^30^ the luminescence of CD@host composites were measured at low temperature (77 K) (Fig. S14†). From which, the *Δ*_EST_ was calculated to be around 0.56 eV, favorable for effective ISC process and spin–orbit coupling. However, such a value was much wider than 0.1 eV, the thermal active delayed fluorescence (TADF) could thus be eliminated.^31^ The afterglow was thus assigned to the RTP process. Furthermore, the origin of RTP from CDs@host composites was systematically investigated. It could be seen from Fig. S15 and S16† that the RTP excitation band overlapped partially with the UV-vis absorption region of C=O/C=N bonds and host matrices. It clearly revealed that in these composites, the two phosphorescent emissive centers mainly stemmed from the C=O/C=N bonds on CDs and IPA/MA constitutive matrices. Moreover, from the FT-IR spectra study, the NH_2_ peak at 3418 cm^−1^ of CDs moved to 3340 cm^−1^ in CDs@IPA, (from 3347 to 3329 cm^−1^ in CDs@MA) indicating strengthened hydrogen bonds between the pivotal NH_2_ of residual MA on the surface of CDs and NH_2_/COOH in MA/IPA matrix were formed during the co-crystallization process (Fig. S17†).^11*e*^ It was well-documented that such hydrogen bonds could effectively construct a rigid environment by immobilizing and restricting vibration and rotation of the emissive species, eventually leading to the effective radiative transition for RTP emission from CDs.^32^ Notedly, the RTP emission from CDs was hardly observed from CDs@IPA under a relatively higher hydrothermal temperature (≥250 °C), because of the over-carbonization,^33^ which consumed too much of emitted species and it was also hard to construct the hydron bond between CDs and IPA molecules. The invariable green RTP emission from the IPA matrix was only thus observed (Fig. S13†). It exactly validated the mechanism of RTP we proposed.

**Fig. 8:**
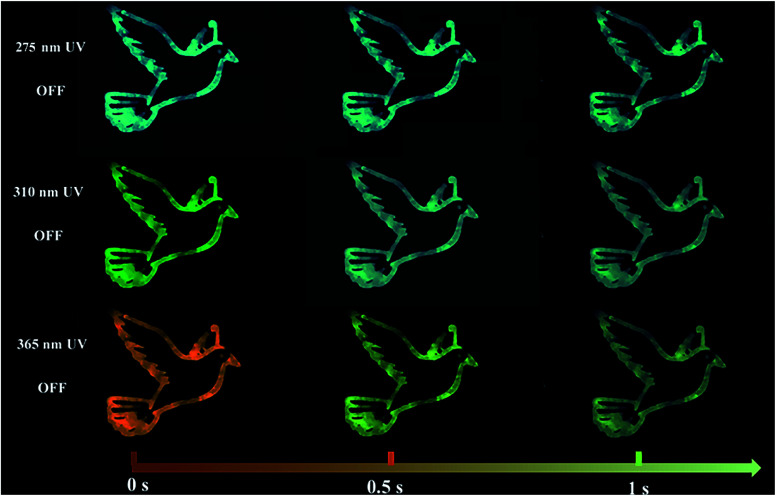
The time-dependent RTP color photographs of dove patterns, recorded with CDs@IPA as the ink, after switching off 275, 310 or 365 nm UV regions.

The outstanding time-dependent evolutive RTP color allowed the prepared CDs@IPA composite to act as a kind of smart anti-counterfeiting material. As shown in Fig. 8, the pattern painted with CDs@IPA exhibited a clear bluish-green “dove” after turning off a 275 nm UV-lamp. Although its brightness was decreasing over time, the persistent invariable bluish-green luminescence still could be observed within 1 s. Especially, for the same pattern with a 365 nm UV lamp switched off, an orange “dove” appeared immediately, then gone through yellow and ultimately turned into a green one. However, when the 365 nm UV source was replaced by a 310 nm analogue, the dynamical RTP colors could also be captured with color variations, but from yellow to green. These phenomena could be easily recognized with naked eyes. Such unique tunable RTP features in the material are thus definitely more difficult to be mimicked or forged and are supposed to be promising candidates for high-level anti-counterfeiting.

## 4. Conclusions

In summary, a facile two-component co-crystallization strategy for the synthesis of time-dependent evolutive RTP colors composites was proposed. The obtained CDs@IPA and CDs@MA were organized by the powerful hydrogen bonds between CDs and the relevant host matrix. They exhibited remarkable dynamic RTP colors from orange *via* yellow to green over 1 s, after ceasing the 365 nm UV-light. Especially, for the CDs@IPA, the ultimate green persistent luminescence lasted for another 6 s, with an average lifetime of 582 ms. The time-dependent RTP emission of such a system could be attributed to the presence of the two primary emissive centers, hydrogen bonds-stabilized low-energy emitted CDs and CD-activated high-energy emitted host matrix (IPA or MA), which displayed distinguishable RTP colors and differentiated lifetimes. The ratios of the two RTP emission bands changed with time. So, that triggered the RTP colors to evolute in real-time during the decay process. However, once the over-carbonization or utilization of other matrices without powerful H-bonds impact or crystallization-induced RTP were carried out, no obvious time-dependent RTP colors were observed. Furthermore, a potential application of CDs@IPA for advanced anti-counterfeiting has been successfully studied. This carbon dot-based co-crystallization strategy with aromatic carboxylic acids or aromatic amines may open a new avenue for the development of time-dependent afterglow materials.

## Conflicts of interest

The authors declare that they have no conflict of interest.

## Supplementary Material

NA-003-D1NA00362C-s001
