# Supplementary material for: A facile co-crystallization approach to fabricate two-component carbon dot composites showing time-dependent evolutive room temperature phosphorescence colors
Source: Nanoscale Adv. 2021 Jul 23;3(17):5053–61. doi: 10.1039/d1na00362c (PMC9417792; doi:10.1039/d1na00362c)
Supplement: NA-003-D1NA00362C-s001 [file NA-003-D1NA00362C-s001.pdf]

Electronic Supplementary Material (ESI) for Nanoscale Advances.
This journal is © The Royal Society of Chemistry 2021

A Facile Co-crystallization Approach to Fabricate Two-component Carbon Dots Composites Showing Time-Dependent Evolutive Room Temperature Phosphorescence Colors

Jian Qu[a], Xin Zhang [a,b,*], Shuyan Zhang[c], Zhongjie Wang[a], Yejian Yu[a], Huajun Ding[a], Zhiyuan Tang[a], Xiangjun Heng[a], Ruiqi Wang[c], Su Jing [c,*]

[a] School of Materials Science and Engineering, Yancheng Institute of Technology, Yancheng 224051, PR China, E-mail: xinzhang@ycit.edu.cn;

[b] School of Chemistry and Chemical Engineering, Southeast University, Nanjing, 211189, China, E-mail: xinzhang@ycit.edu.cn;

[c] School of Chemistry and Molecular Engineering, Nanjing Tech University, Nanjing 211816, P.R. China. E-mail: sjing@njtech.edu.cn;

[*] Corresponding author

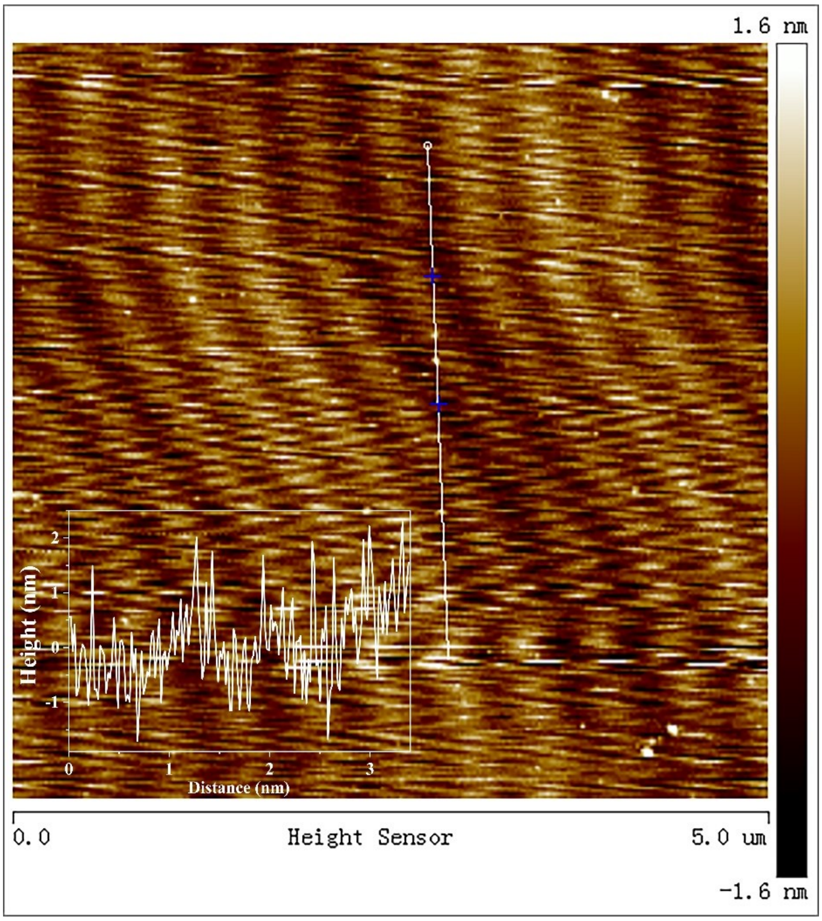

Fig. S1 AFM image of CDs

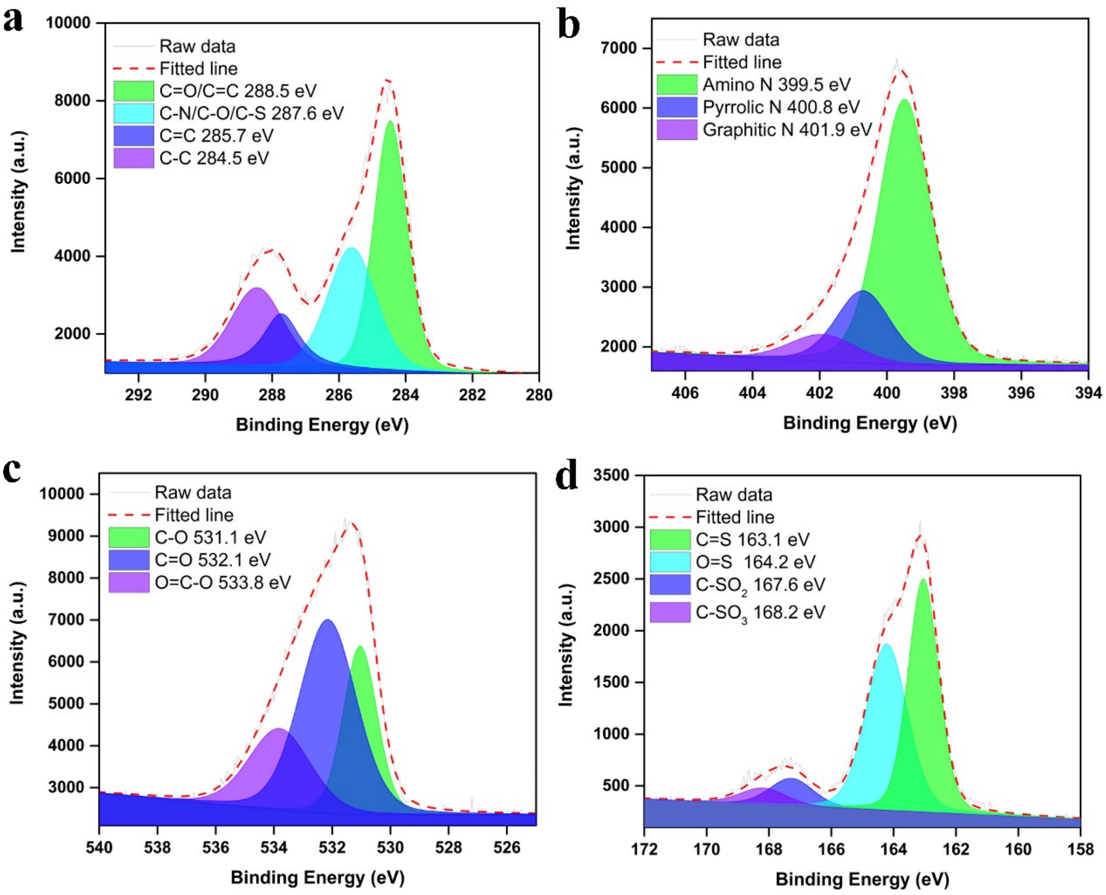

Fig. S2 a) C1s spectrum b) N1s spectrum c) O1s spectrum d) S2p spectrum of CDs, respectively.

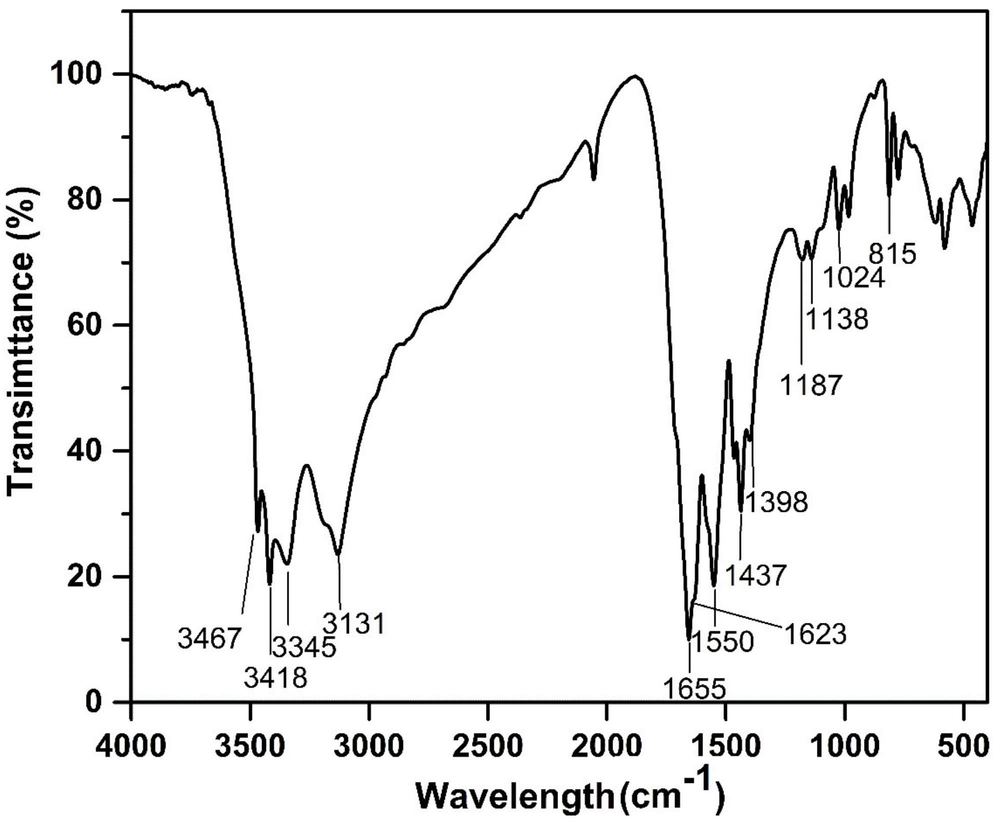

Fig. S3 FT-IR spectrum of CDs.

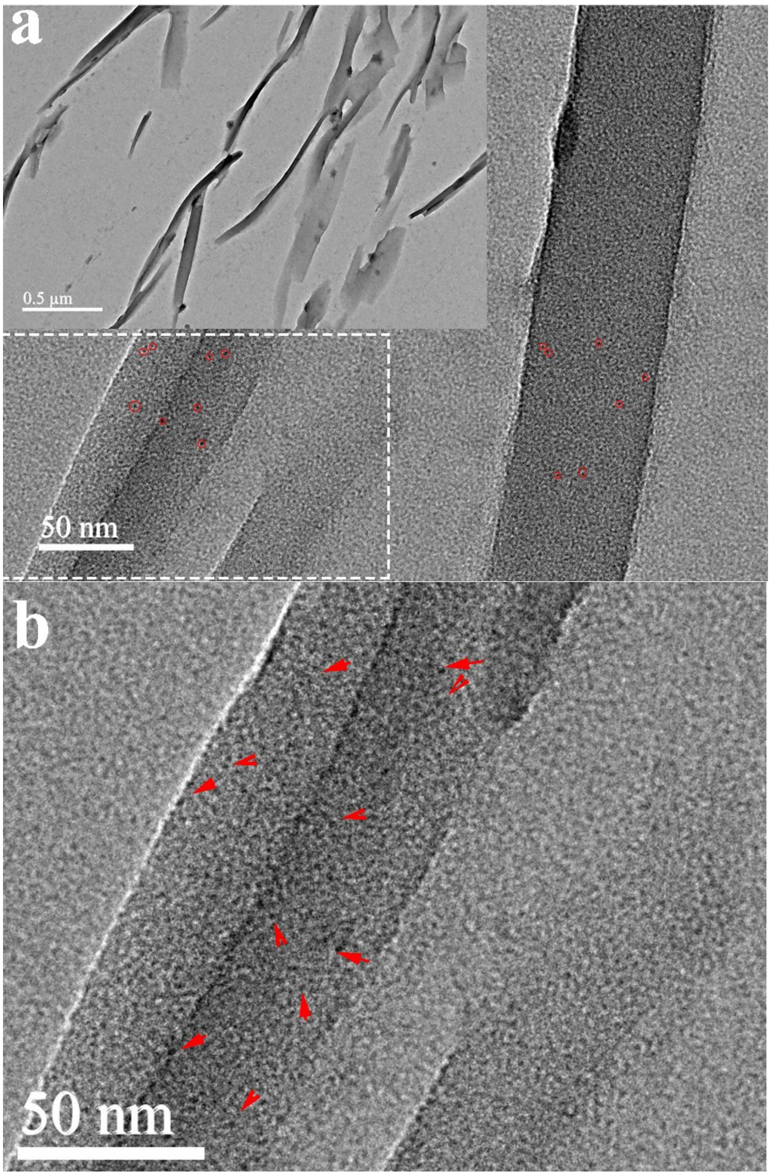

Fig. S4 TEM images of CDs@IPA under 0.5 μm and 50 nm scale bar. b) The local TEM image of CDs@IPA.

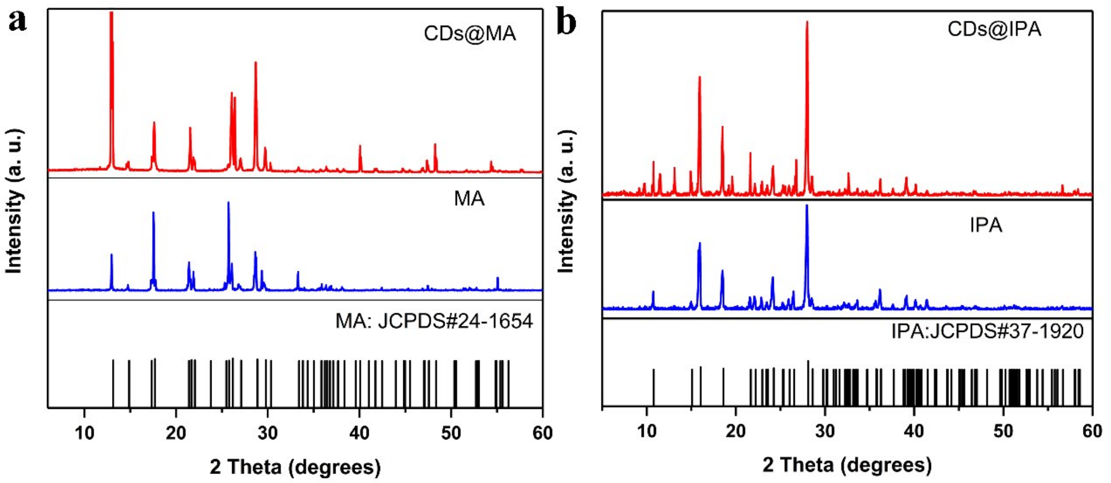

Fig. S5 a) XRD pattern of the CDs@MA, MA and MA XRD card. b) XRD pattern of the CDs@IPA, IPA and IPA XRD card.

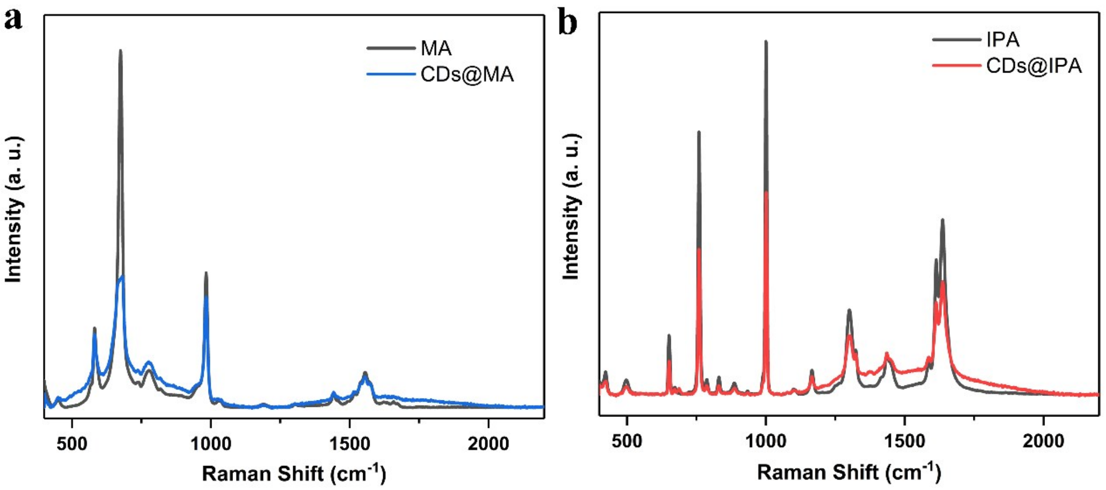

Fig. S6 a) Raman spectra of CDs@MA and MA. b) Raman spectra of CDs@IPA and IPA.

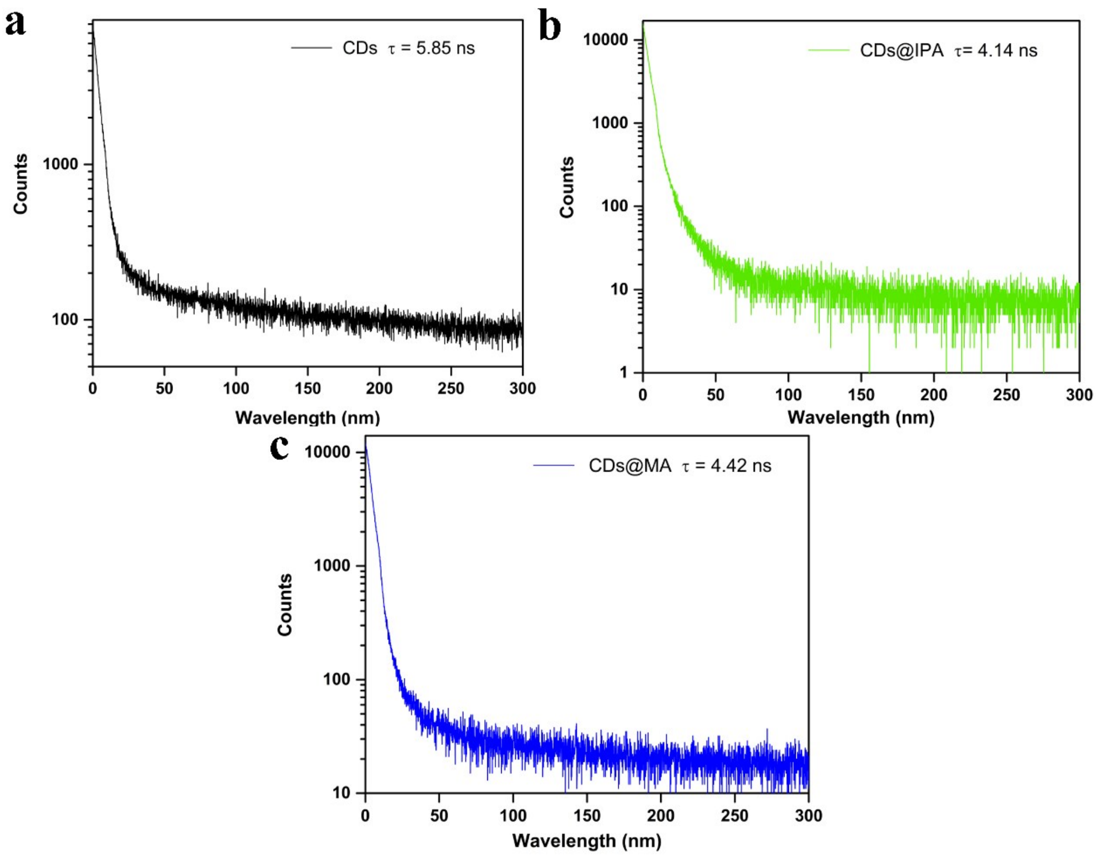

Fig. S7 FL decay curves of a) CDs, b) CDs@IPA and c) CDs@MA

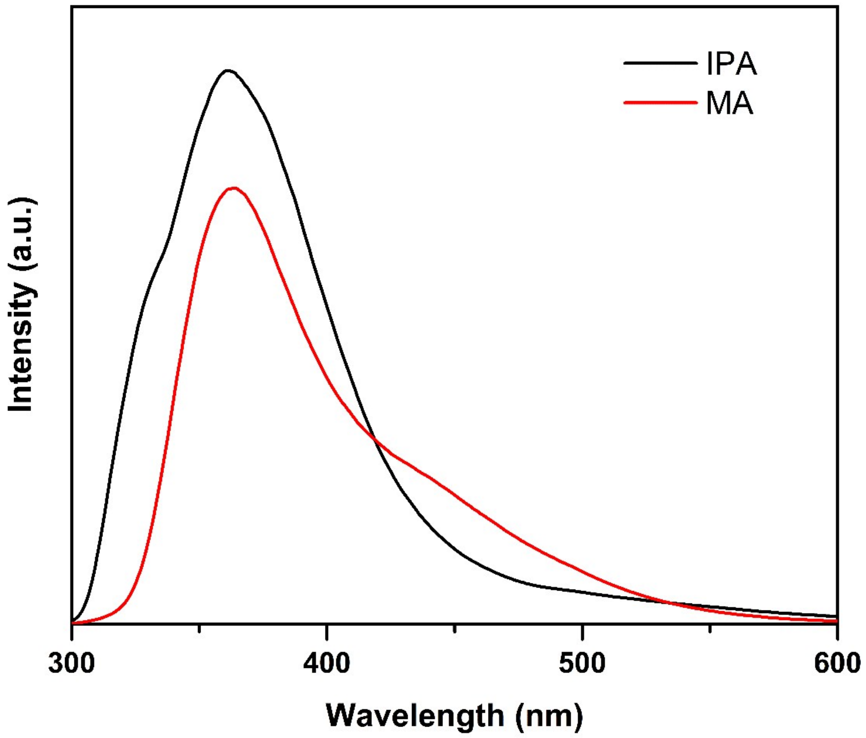

Fig. S8 The FL emission spectra of IPA and MA (λ_{ex}=280 nm).

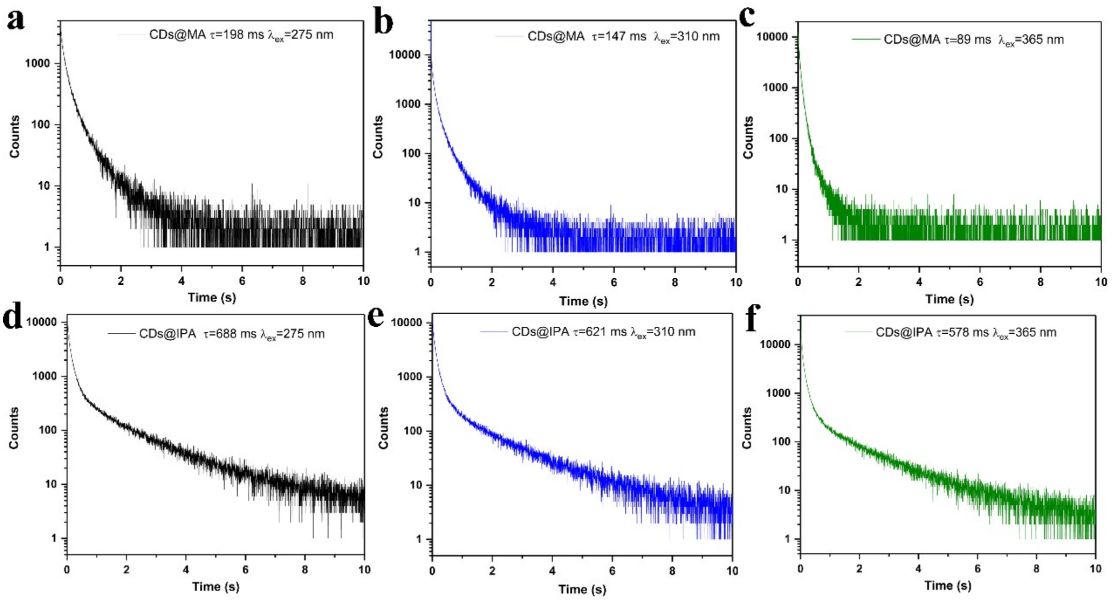

Fig. S9 RTP decay profiles of CDs@MA under excitation wavelength at a)275nm, b) 310 nm and c) 365 nm. RTP decay profiles of CDs@IPA under excitation wavelength at d)275nm, e) 310 nm and f) 365 nm.

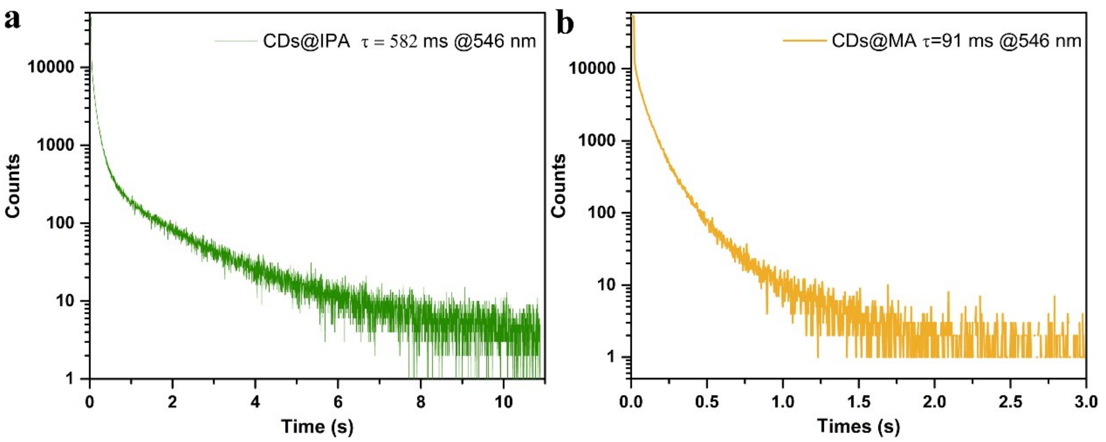

Fig. S10 RTP decay profiles of a) CDs@IPA and b) CDs@MA.

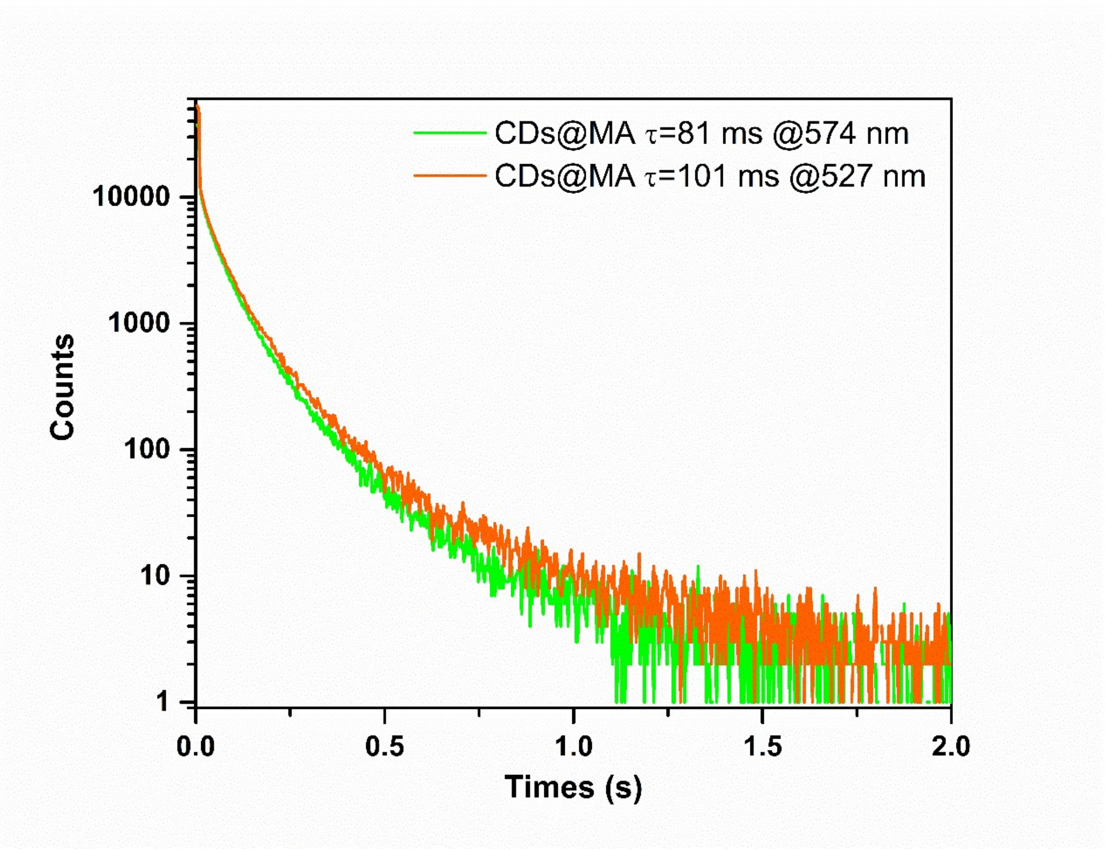

Fig. S11 Corresponding RTP decay profiles of CDs@MA (λ_{ex} = 360 nm).

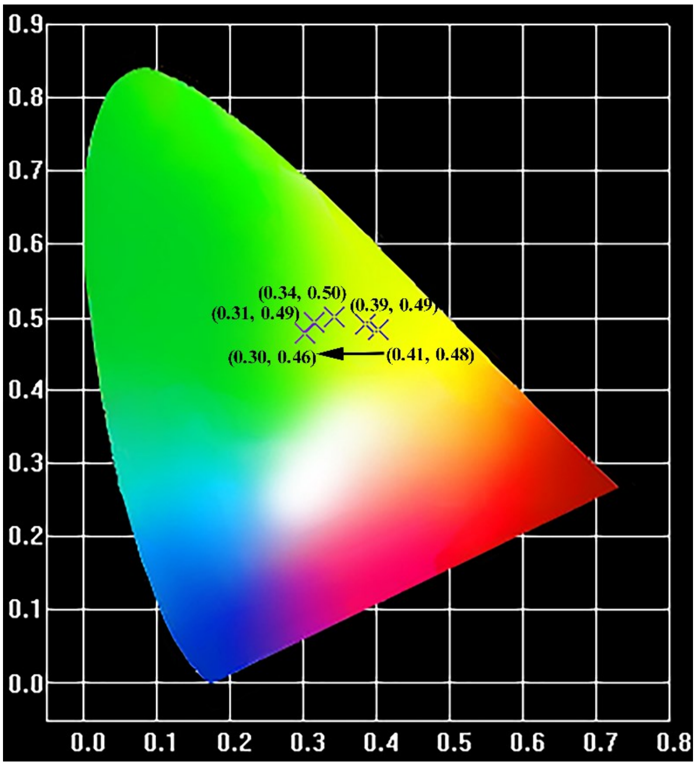

Fig. S12 CIE coordinates of time-dependent color of CDs@MA.

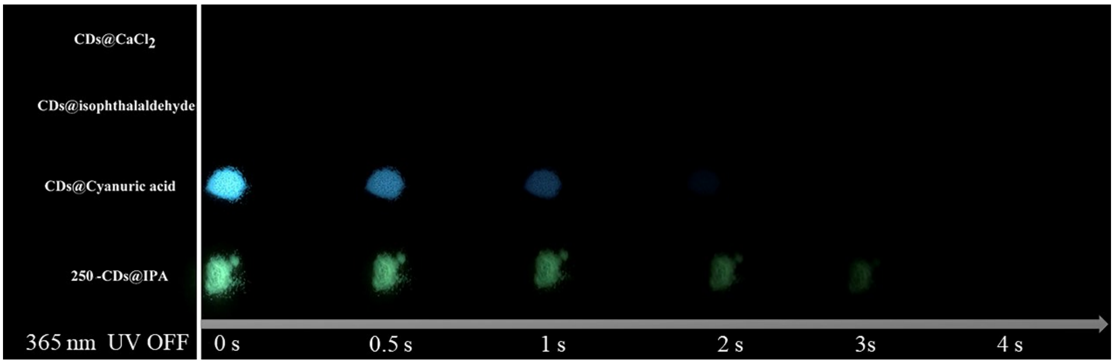

Fig. S13 Phtotographs of $CDs@CaCl_2$, CDs@isophthalaldehyde, CDs@cyanuric acid and 250℃-CDs@IPA.

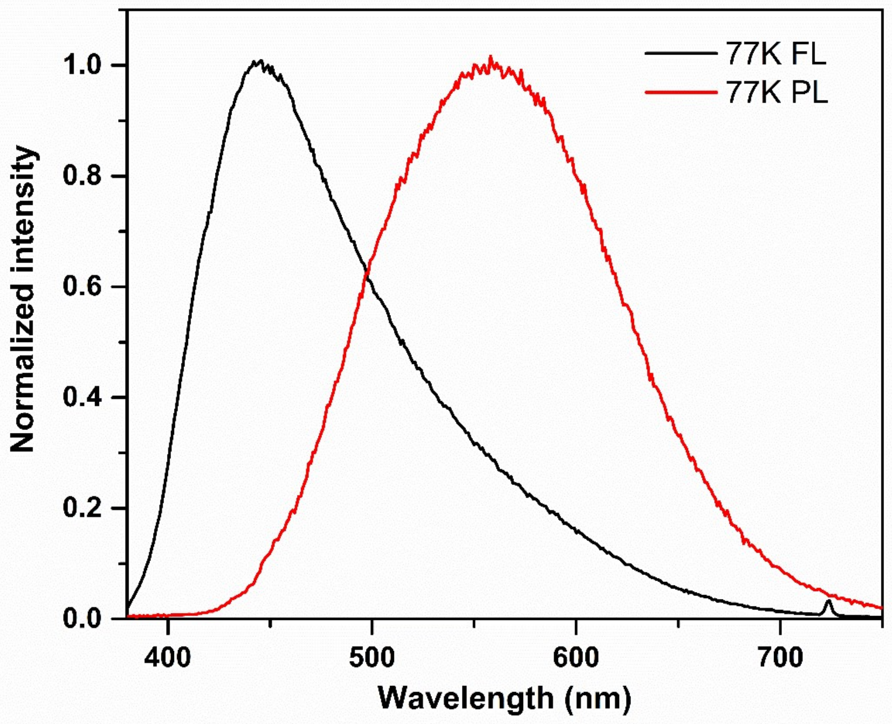

Fig. S14 Normalized fluorescence and phosphorescence spectra of CDs@IPA at 77 K (λ_{ex} = 360 nm).

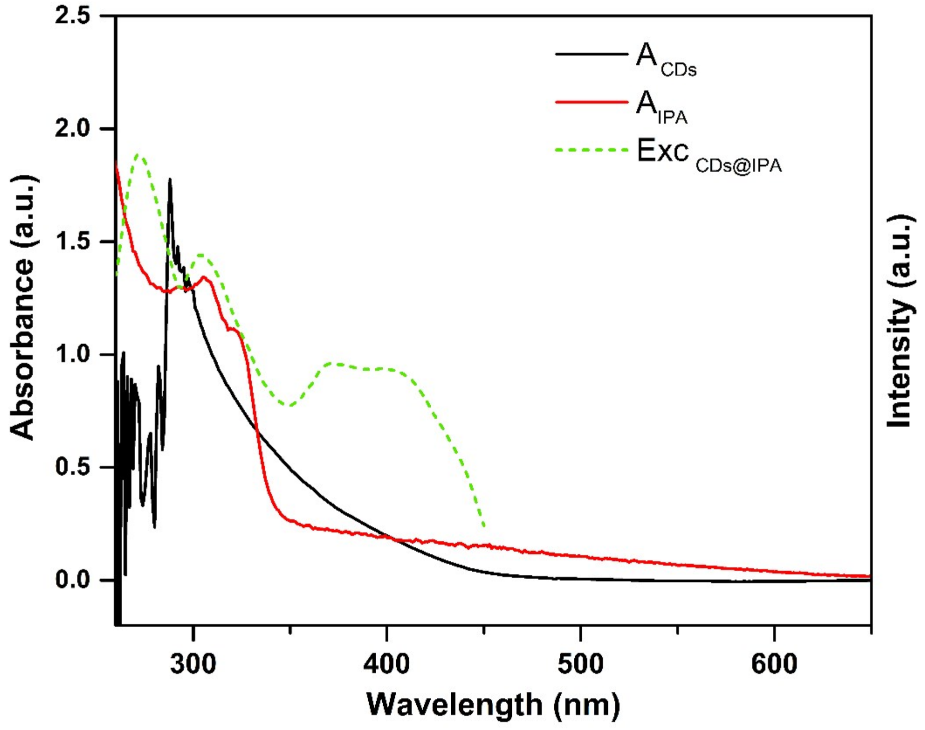

Fig. S15 UV-vis absorbance spectra of CDs and IPA matrix, and the RTP excitation spectrum of CDs@IPA at 546 nm.

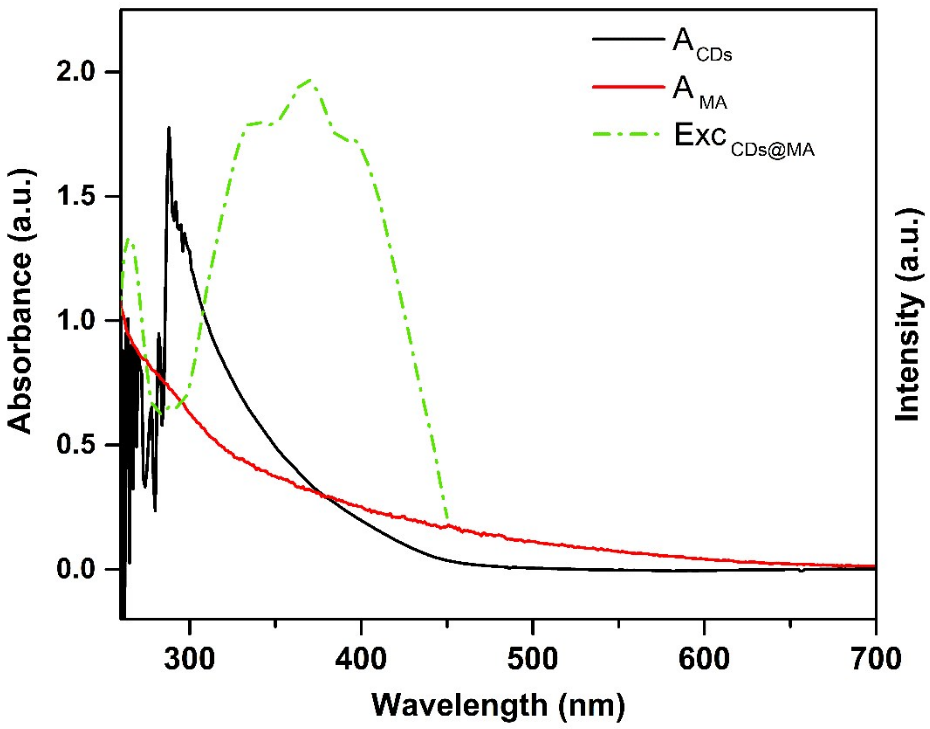

Fig. S16 UV-vis absorbance spectra of CDs and MA matrix, and the RTP excitation spectrum of CDs@MA at 546 nm.

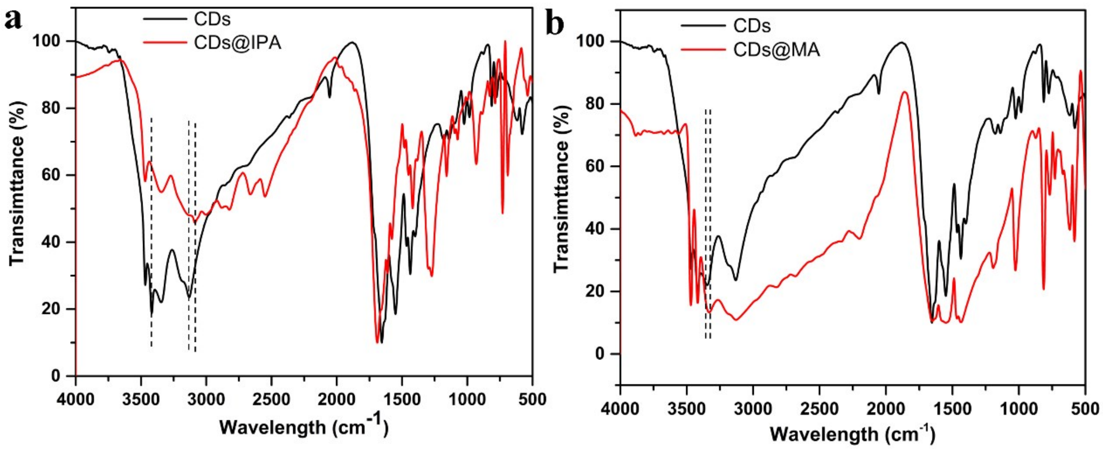

Fig. S17 FT-IR spectrum of CDs@IPA and CDs@MA.

Table S1. The RTP decay profiles of the CDs@IPA and CDs@MA at different emission wavelength.

Composite	λ_{em} (nm)	τ_1 (ms)	B_1 (%)	τ_2 (ms)	B_2 (%)	τ_3 (ms)	B_3 (%)	τ_{avg} (ms)
CDs@IPA	546	179.85	39.00	1509.00	32.90	50.87	28.10	582
	532	190.75	36.26	1552.57	39.13	53.65	24.61	686
	578	146.34	44.51	4.12	32.19	1366.61	23.30	395
CDs@MA	546	18.47	13.97	67.39	71.73	207.55	18.64	91
	527	20.35	14.70	76.34	66.25	244.08	19.05	101
	574	16.16	12.71	65.16	68.75	188.72	18.54	81
